# ParAquaSeq, a Database of Ecologically Annotated rRNA Sequences Covering Zoosporic Parasites Infecting Aquatic Primary Producers in Natural and Industrial Systems

**DOI:** 10.1111/1755-0998.14099

**Published:** 2025-03-15

**Authors:** Silke Van den Wyngaert, Slawek Cerbin, Laura Garzoli, Hans‐Peter Grossart, Alena S. Gsell, Alexandra Kraberg, Cécile Lepère, Sigrid Neuhauser, Miloš Stupar, Andrea Tarallo, Michael Cunliffe, Claire Gachon, Ana Gavrilović, Hossein Masigol, Serena Rasconi, Géza B. Selmeczy, Dirk S. Schmeller, Bettina Scholz, Natàlia Timoneda, Ivana Trbojević, Elżbieta Wilk‐Woźniak, Albert Reñé

**Affiliations:** ^1^ Department of Biology University of Turku Turku Finland; ^2^ Department of General Zoology, Faculty of Biology Adam Mickiewicz University Poznań Poland; ^3^ Water Research Institute (CNR‐IRSA) National Research Council of Italy Verbania Italy; ^4^ Department of Plankton and Microbial Ecology Leibniz Institute of Freshwater Research and Inland Fisheries (IGB) Stechlin Germany; ^5^ Department of Biochemistry and Biology Potsdam University Potsdam Germany; ^6^ Department of Environmental Biology, Institute of Environmental Sciences (CML) University of Leiden Leiden the Netherlands; ^7^ Department of Aquatic Ecology Institute of Ecology (NIOO‐KNAW) Wageningen the Netherlands; ^8^ Alfred Wegener Institute Helmholtz Centre for Polar and Marine Research Bremerhaven Germany; ^9^ CNRS, Laboratoire Microorganismes: Génome et Environnement, Université Clermont Auvergne Clermont‐Ferrand France; ^10^ Institute of Microbiology Universität Innsbruck Innsbruck Austria; ^11^ Institute of Botany and Botanical Garden “Jevremovac”, Faculty of Biology University of Belgrade Belgrade Serbia; ^12^ National Research Council (CNR), Institute of Research on Terrestrial Ecosystems (IRET) Lecce Italy; ^13^ Marine Biological Association, The Laboratory Plymouth UK; ^14^ School of Biological and Marine Sciences University of Plymouth Plymouth UK; ^15^ Muséum National d'Histoire Naturelle, UMR 7245 CNRS, CP 64 Paris France; ^16^ University of Zagreb Faculty of Agriculture Zagreb Croatia; ^17^ Université Savoie Mont Blanc, INRAE, CARRTEL Thonon les Bains France; ^18^ Research Group of Limnology, Center for Natural Science University of Pannonia Veszprém Hungary; ^19^ HUN‐REN‐PE Limnoecology Research Group Veszprém Hungary; ^20^ Centre de Recherche sur la Biodiversité et l'Environnement (CRBE), Université de Toulouse, CNRS, IRD, Toulouse INP, Université Toulouse 3 – Paul Sabatier (UT3) Toulouse France; ^21^ BioPol ehf Skagaströnd Iceland; ^22^ Department of Biologia Marina i Oceanografia Institut de Ciències del Mar (ICM‐CSIC) Barcelona Catalonia Spain; ^23^ Institute of Nature Conservation Polish Academy of Sciences Kraków Poland

**Keywords:** aquatic plants, chytrids, fungal parasites, macroalgae, metabarcoding, microalgae

## Abstract

Amplicon sequencing tools such as metabarcoding are commonly used for thorough characterisation of microbial diversity in natural samples. They mostly rely on the amplification of conserved universal markers, mainly ribosomal genes, allowing the taxonomic assignment of barcodes. However, linking taxonomic classification with functional traits is not straightforward and requires knowledge of each taxonomic group to confidently assign taxa to a given functional trait. Zoosporic parasites are highly diverse and yet understudied, with many undescribed species and host associations. However, they can have important impacts on host populations in natural ecosystems (e.g., controlling harmful algal blooms), as well as on industrial‐scale algae production, e.g. aquaculture, causing their collapse or economic losses. Here, we present ParAquaSeq, a curated database of available molecular ribosomal sequences belonging to zoosporic parasites infecting aquatic vascular plants, macroalgae and photosynthetic microorganisms, i.e. microalgae and cyanobacteria. These sequences are aligned with ancillary data and other information currently available, including details on their hosts, occurrence, culture availability and associated bibliography. The database includes 1131 curated sequences from marine, freshwater and industrial or artificial environments, and belonging to 13 different taxonomic groups, including Chytridiomycota, Oomycota, Phytomyxea, and Syndiniophyceae. The curated database will allow a comprehensive analysis of zoosporic parasites in molecular datasets to answer questions related to their occurrence and distribution in natural communities. Especially through meta‐analysis, the database serves as a valuable tool for developing effective mitigation and sustainable management strategies in the algae biomass industry, but it will also help to identify knowledge gaps for future research.

## 1 Introduction

**FIGURE 1:**
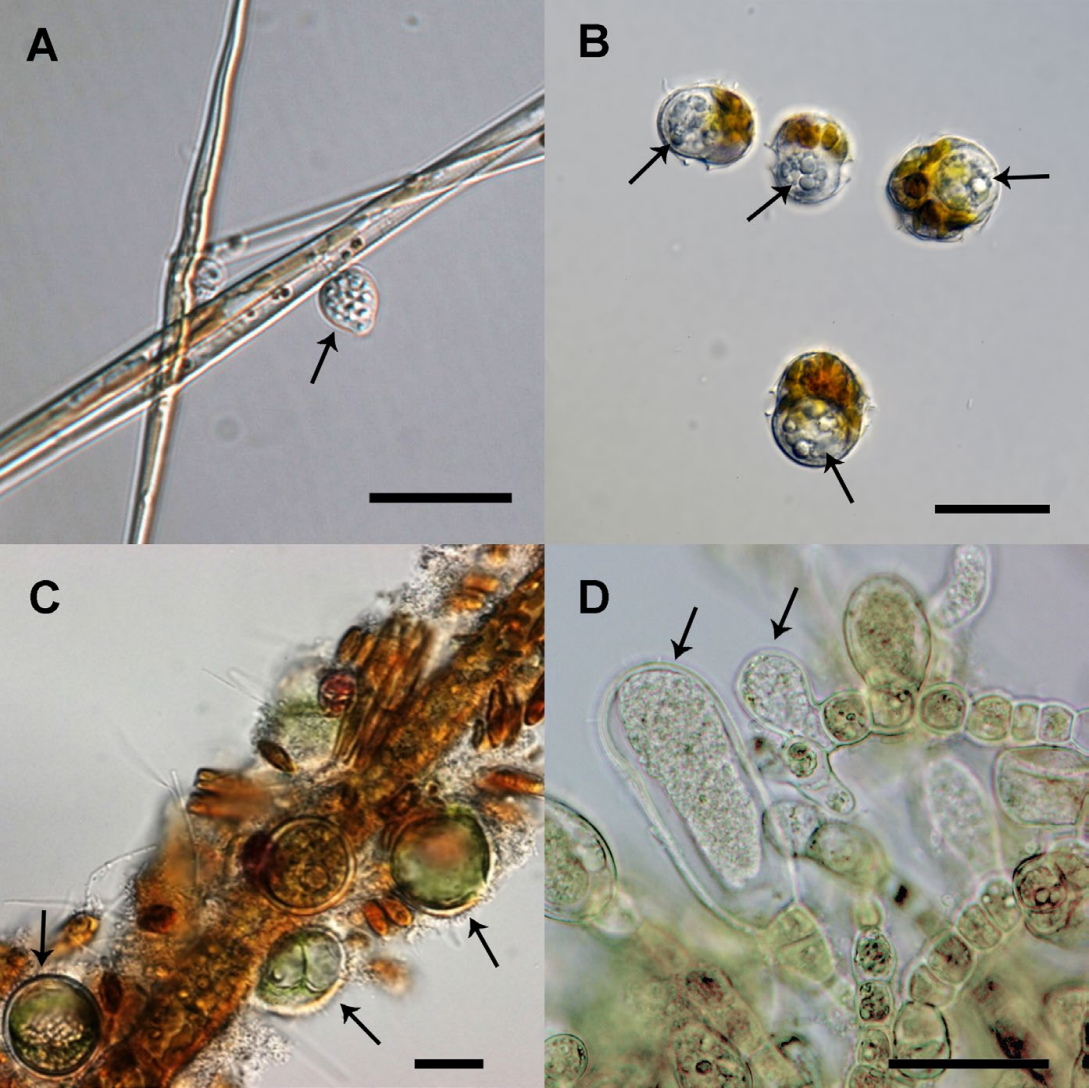
Illustration of zoosporic parasites infecting aquatic primary producers. (A) Freshwater diatom *Ulnaria* sp. (former *Synedra* sp.) infected by the chytrid *Zygophlyctis planktonicum*. (B) Marine dinoflagellate *Alexandrium minutum* infected by the perkinsean endoparasite *Parvilucifera sinerae*. (C) Red macroalgae *Palmaria palmata* infected by the oomycete *Olpidiopsis palmariae* (modified from Badis et al. 2019). (D) Brown macroalgae *Macrocystis* sp. infected by *Maullinia ectocarpii*. Arrows indicate the parasite. Scale bars = 20 μm.

Aquatic primary producers represent a key component of aquatic ecosystems as they form the basis of most aquatic food webs and provide over half of the planet's oxygen production and net carbon fixation (Woodward 2007). Moreover, they are of increasing bioeconomic interest as renewable resources for food, feed (Kumar et al. 2019), energy, refined products such as pharma‐ and nutraceuticals (Khan et al. 2018), and as wastewater remediation agents (Araújo et al. 2021). However, they are vulnerable to infections caused by parasites, in particular microeukaryotic zoosporic fungal and fungal‐like parasites. Zoosporic parasites are a polyphyletic group of eukaryotes with similar morphological traits, comprising species from various taxonomic phyla, including Fungi, Oomycota, Alveolata, or Phytomyxea (Figure 1). They are characterised by their mobile, infective life stage (zoospore), which is incapable of nutrient uptake, has a limited off‐host lifetime, and requires attachment to their host to develop feeding and reproductive structures. This definition excludes other organisms that feed on prey following a phagotrophic strategy. Zoosporic parasites can be either facultative or obligate parasites of a wide variety of substrates including aquatic primary producers where they form epi‐ or endobiotic infections. Although molecular sequence data on zoosporic organisms is increasingly available, it remains largely disconnected from their ecological function (Van den Wyngaert et al. 2022). This knowledge gap hampers reliable identification and impact assessment of zoosporic parasites in ecological studies and on the biomass production of aquatic primary producers (Rasconi et al. 2022).

In aquatic primary producers, zoosporic parasite epidemics can reach high infection prevalence rates and hence drastically regulate population size and community dynamics in freshwater (Ibelings et al. 2011; Rasconi et al. 2012; Van den Wyngaert et al. 2022) and coastal environments (Sparrow 1969; Chambouvet et al. 2008). Zoosporic parasites have been reported to influence key ecological processes, including succession of phytoplankton communities (Van Donk and Ringelberg 1983; Sime‐Ngando et al. 2015) and regulation of host genetic diversity (Gsell et al. 2013). Furthermore, zoosporic parasites can promote the trophic transfer of organic matter locked in inedible phytoplankton species through the production of edible and nutrient‐rich zoospores, i.e. the “mycoloop” (Grami et al. 2011; Kagami et al. 2014) and through tighter coupling of the microbial food web, i.e. the “fungal shunt” (Klawonn et al. 2021). In industrial algal biomass production, contaminations with zoosporic parasites can lead to production disruption, requiring mechanical and chemical disinfection of production systems, incurring costs in terms of production losses and system operation expenditure (Carney and Lane 2014; Asatryan et al. 2019).

Identification of zoosporic parasites has long been based exclusively on microscopic observations of the morphology of their sporangia (a pouch‐like structure in which zoospores are formed and stored) in live plankton samples (Karling 1942; Sparrow 1960; Johnson and Sparrow 1961), resulting in detailed descriptions of their morphological features and designation of host species (Ingold 1940; Canter 1949). However, the lack of reliable species‐specific morphological features makes species‐level identification of zoosporic parasites in environmental samples by light and fluorescence microscopy almost impossible (Miller 1968). Using electron microscopy to explore the ultrastructural cytology of, for instance, chytrid zoospores may overcome these limitations (e.g., Letcher et al. 2008; Seto et al. 2020). However, zoospores are rarely detected in environmental plankton samples and, hence, ultrastructural characterisation of zoospores for reliable identification (Barr 1980; Longcore 1995; Hanic et al. 2009; Letcher and Powell 2014) are generally not feasible in traditional monitoring for zoosporic infections.

With the surge in metabarcoding amplicon sequencing of environmental samples, the availability of 18S, 28S rRNA, and ITS sequences of fungal and fungal‐like organisms is increasing almost exponentially (Schoch et al. 2012). However, amplicon sequencing does not (yet) provide functional annotation of the trophic strategy in zoosporic taxa, and host identity often remains undocumented. Hence, using sequence data to assess the impact of zoosporic parasites and their ecological roles and consequences remains a major challenge, although metatranscriptomics approaches overcoming this limitation are being developed (Monjot et al. 2023). Recently, script‐based bioinformatic tools linking fungal taxonomy and molecular data to ecological guilds and functions have been developed (Nguyen et al. 2016; Põlme et al. 2020; Krivonos et al. 2023) but their use remains challenging for non‐specialists. A FAIR (findable, accessible, interoperable, and reusable), GUI (graphical user interface) based and curated sequence database for reliable identification of all zoosporic parasites (i.e., not just fungi) of aquatic primary producers, allowing linking to their ecological role and host range, is still missing. The lack of such a database hampers progress in (a) assessing the occurrence and dynamics of parasitic lifestyle, biogeography, and eco‐evolutionary research and (b) developing early detection methods for biomass production facilities.

Here, we address this knowledge gap by providing a database, PARasite AQUAtic SEQuences—ParAquaSeq, gathering molecular ribosomal sequences belonging to confirmed or putative zoosporic parasites of aquatic primary producers (i.e., microalgae, cyanobacteria, macroalgae, and aquatic vascular plants) annotated with their associated metadata, such as confirmed host groups, culture availability, location of isolation, or habitat.

## 2 Materials and Methods

Firstly, we downloaded all sequences belonging to taxonomic groups known to contain zoosporic parasites infecting photosynthetic microorganisms, i.e. microalgae and cyanobacteria, macroalgae, and aquatic vascular plants (macrophytes) available in NCBI, and their associated metadata. Given that the functional traits of organisms are not labelled in NCBI records, a bibliographic search was performed to select taxonomic groups known to include zoosporic parasites of primary producers. We developed a script for querying the NCBI database using the R environment v 4.1.0 (R Core Team 2023), available at GitHub (https://github.com/ParAqua‐COST/ParAquaSeq_Repository). In brief, we modified the R package ‘rentrez’ (v. 1.2.3, Winter 2017), which provides functions to use Entrez Utilities via REST API and gather the data from multiple NCBI databases. The script extracts all required information and creates a raw database ready for further manual verification. The queries were constructed for all groups to obtain sequences between 500 and 20,000 bp from the 18S, 5.8S, 28S rRNA, and ITS. We targeted only ribosomal gene sequences, as they are the universal markers commonly used by metabarcoding techniques to determine environmental diversity, including fungi, fungal‐like organisms, and protists, i.e. potential zoosporic parasites. All queries used can be found at the ParAquaSeq GitHub repository. The queries constructed for several groups excluded some taxa known for not being parasites or for not being parasites of the target host group (aquatic primary producers) to avoid including those entries in the initial search. Access to NCBI was conducted between March–April 2023, depending on the taxonomic group, except for the searches done for *Pirsonia*, *Pseudopirsonia*, and *Cryothecomonas*, performed in April 2024. In this case, sequences published after March 2023 were not considered to prevent incongruences with previously accessed taxonomic groups.

**FIGURE 2:**
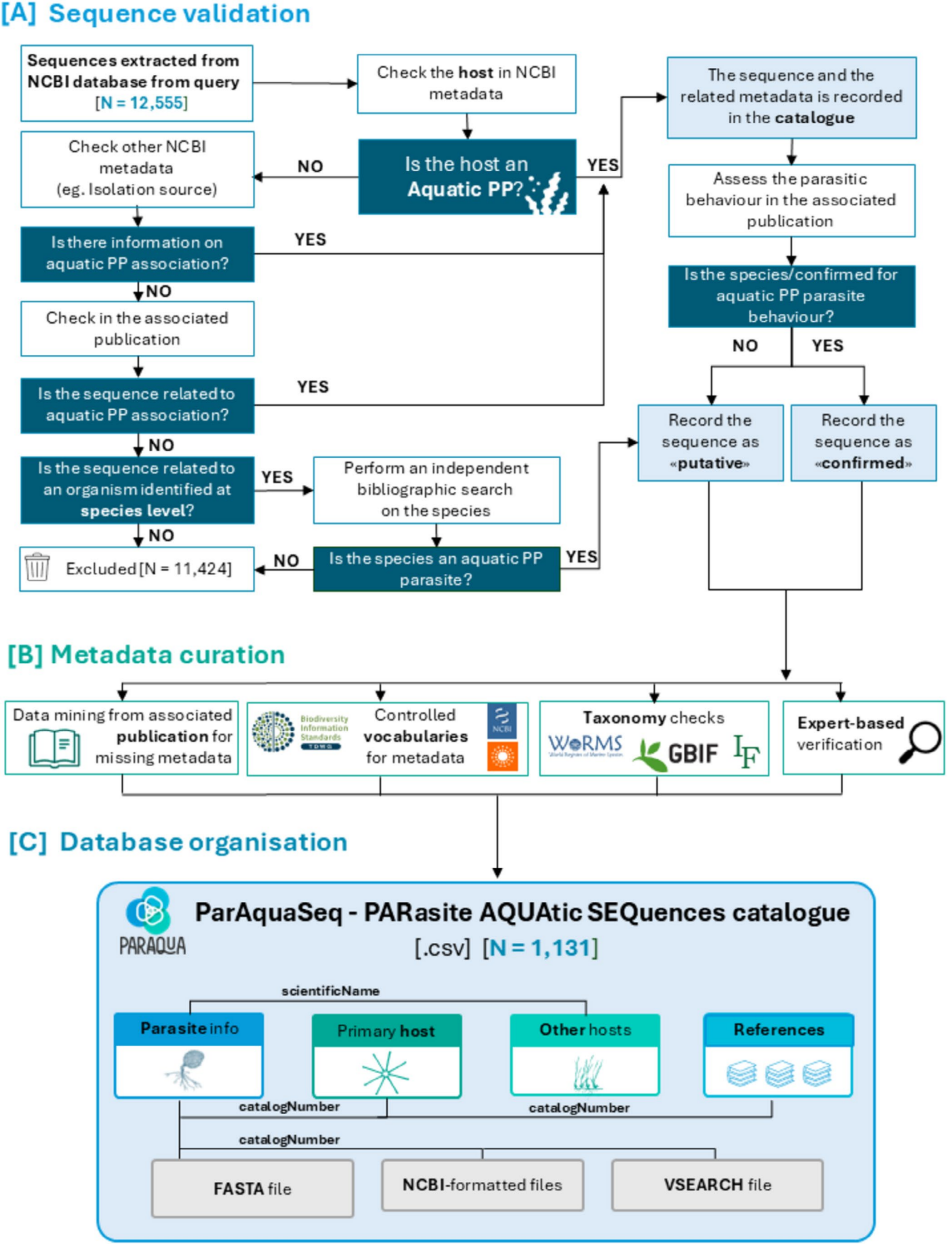
Summary of the workflow for the creation of ParAquaSeq—PARasite AQUAtic SEQuences database for sequences of zoosporic parasites infecting aquatic primary producers (PP). (A) Decision process for sequence inclusion/exclusion. (B) Summary of metadata curation, including reference to online repositories consulted. (C) Organisation of the ParAquaSeq database including table connectors.

To ensure consistency in the manual curation of sequences across taxonomic groups, we followed a standardised stepwise protocol outlined in the workflow diagram (Figure 2). A thorough manual verification process for each entry was conducted to determine if the sequence belonged to a parasite or a putative parasite of aquatic photosynthetic microorganisms, macroalgae, or macrophytes. Initially, we checked for the availability of host and/or isolation source information in the NCBI record, ensuring correspondence with aquatic photosynthetic taxa. Where no host information was provided, we consulted the associated publication. If the publication also lacked host information, we performed an independent bibliography search for entries with full species names. This search aimed to determine whether the species had been documented elsewhere as a parasite of an aquatic primary producer. It involved consulting the chytrid species catalogue compiled by Joyce Longcore (https://czeum.herb.lsa.umich.edu/bibliography‐of‐dr‐joyce‐e‐longcore/) (only applicable to zoosporic fungi) and using Google Scholar with the combined search string: parasite, parasitic, and the species name. An entry was categorised as a confirmed parasite only if it was explicitly described as such in the associated publication and/or GenBank information. Entries were retained and labelled as “putative” only if the same species name was associated elsewhere in the literature as a parasite in conjunction with an algal host source in relation to the species name without direct description of observed infections. All other sequences were removed from the datasets. For all entries categorised as “parasite” or “putative parasite” we consulted the associated publication to gather additional relevant information not present in NCBI metadata, like habitat, location, or alternative hosts. To ensure the consistency of the data aggregated from different resources, both the variable titles, i.e., the headers of the tables, and the values that each variable can assume were controlled and harmonised. To enhance the reusability of the data and the interoperability with other systems, we employed controlled and recognised terminologies whenever possible, mainly Darwin Core (Darwin Core Maintenance Group 2023). The database's taxonomy was curated primarily using the Global Biodiversity Information Facility Taxonomic Backbone (GBIF Secretariat 2023), World Register of Marine Species (WoRMS Editorial Board 2024), and Index Fungorum (www.indexfungorum.org). In some cases, we adopted a unified classification for consistency between members of the same taxonomic groups, e.g., Alveolata, due to differing classification at higher taxonomic levels among the different taxonomic backbones used. That classification thus differed from the original GBIF and WoRMS taxonomic backbones, and taxa not yet included in these databases were classified by consulting the literature. Subsequently, experts in specific groups manually verified the taxonomy to ensure its accuracy.

The database, ParAquaSeq, includes different datasets, all of them publicly available at the GitHub repository (https://github.com/ParAqua‐COST/ParAquaSeq_Repository). Sequence datasets hosted on the GitHub repository are formatted to suit diverse end users, being offered in three distinct formats: (1) FASTA format for general use, (2) FASTA format for VSEARCH use, (3) NCBI formatted specifically for blast queries. General FASTA and VSEARCH formats can be used for the taxonomic assignment of metabarcoding amplicons under DADA2 or RDP naive Bayesian classifier environments. Detailed usage instructions are also available on GitHub.

Furthermore, following the above analysis, four interconnected tables were obtained: one primary table summarising the taxonomy of the parasites and the sequence‐related data; one with the information of the primary host, i.e. the host reported on the NCBI entry; one with other known hosts; and one for the associated literature. Tables summarising each variable, its definition, and the standard terminology used are also available at the GitHub repository.

## 3 Results

### 3.1 The ParAquaSeq Database

**FIGURE 3:**
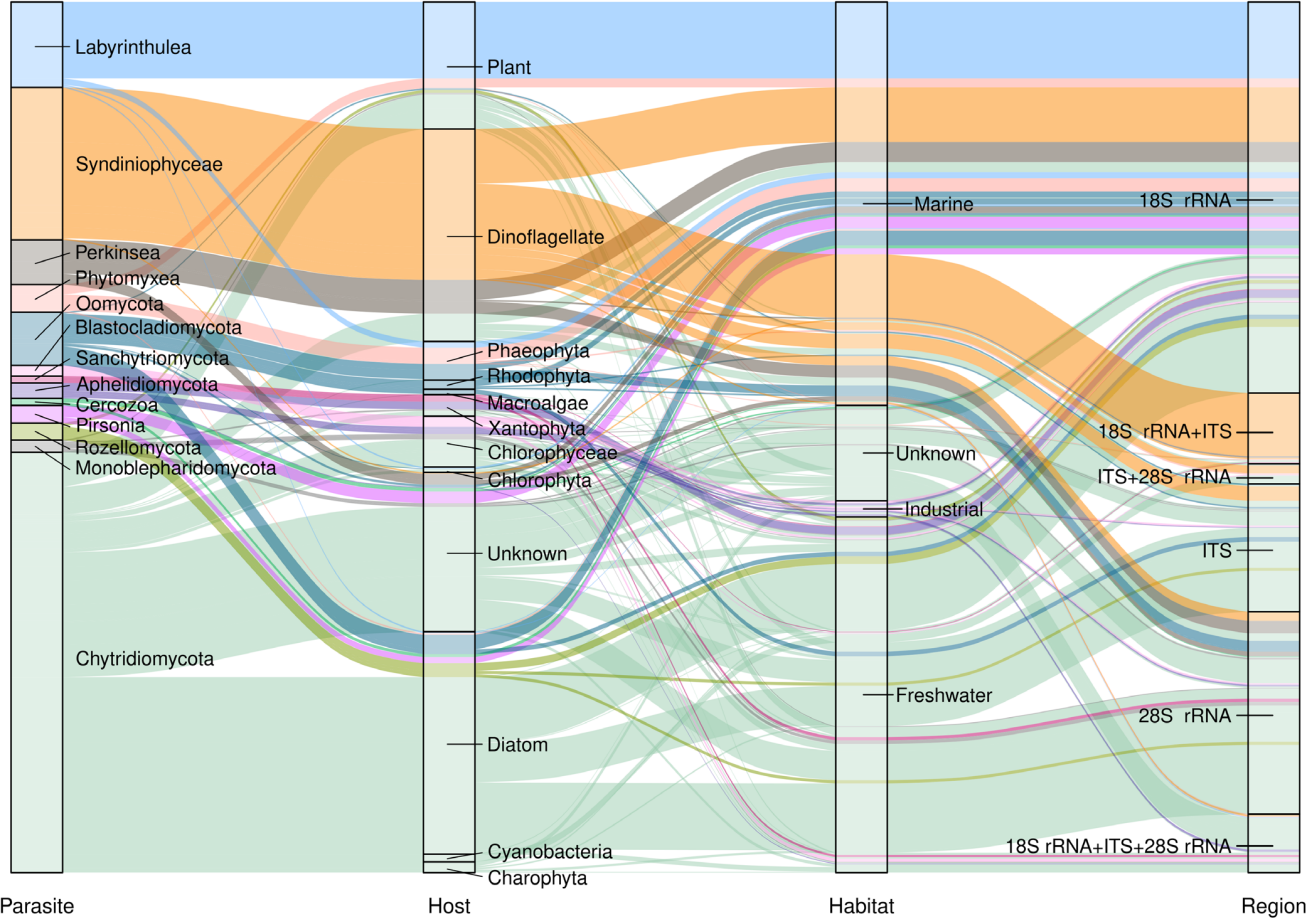
Sankey diagram showing the correspondence between parasitic group, host group, aquatic habitat, and ribosomal region for the sequences included in ParAquaSeq (*n* = 1131).

After the curation process, a total of 1131 sequences were determined as belonging to parasites of cyanobacteria, microalgae, macroalgae, and aquatic vascular plants, including confirmed and putative cases (Figure 3; Table 1). The sequences represented 13 taxonomic groups, comprising Chytridiomycota, Monoblepharidomycota, Blastocladiomycota, Sanchytriomycota, Rozellomycota, Aphelidiomycota (Fungi), Syndiniophyceae, Perkinsea (Alveolata), Phytomyxea, the Cercozoa genera *Cryothecomonas* and *Pseudopirsonia* (Rhizaria), Oomycota, Labyrinthulomycota, and *Pirsonia* (Stramenopiles). The remaining parasitic groups included in the original search yielded no sequences linked to aquatic primary producers. The sequences included in ParAquaSeq covered the different genes of the ribosomal operon, either completely or partially. Five hundred and eight sequences corresponded to 18S rRNA sequences, including sequences of all studied groups. One hundred and sixty‐six sequences corresponded to ITS, in this case exclusively representing fungal groups, Syndiniophyceae, and Oomycota. Two hundred and sixty‐three sequences corresponded to 28S rRNA, mainly represented by groups such as Oomycota, Syndiniophyceae, and Perkinsea. Additionally, some sequences included more than one gene, both completely and partially. In this case, 94 sequences included the 18S rRNA and ITS, mostly Syndiniophyceae; 26 sequences included the ITS and 28S rRNA, mostly Syndiniophyceae and Chytridiomycota; and finally, 74 sequences included all three regions, mostly belonging to Chytridiomycota. Up to 530 sequences corresponded to Chytridiomycota (Fungi), being the group with the highest number of parasite taxa and sequences in the survey (Figure 3; Table 1). It was followed by Syndiniophyceae (Alveolata), which included 198 sequences representing a reduced number of species corresponding mainly to the genus *Amoebophrya*. The parasite group Labyrinthulomycetes (Stramenopiles) was represented by 111 sequences. Oomycota were represented by 69 sequences; 58 corresponded to Perkinsea, and 36 to Phytomyxea. The remaining groups included only a few sequences each.

**TABLE 1:** Overview of sequences represented in the ParAquaSeq database by taxonomic group. Information on the number of known genera and species, their trophic lifestyles, the ribosomal markers, the host groups, and the habitats for each parasite group is included.

Parasite	Chytridiomycota	Aphelidiomycota	Rozellomycota	Monoblepharidomycota	Sanchytriomycota	Blastocladiomycota	Oomycota	Labyrinthulomycota	*Pirsonia*	Phytomyxea	*Pseudopirsonia* and *Cryothecomonas*	Perkinsea	Syndiniophyceae	Total
Diversity														
Sequences	530	20	22	32	9	14	69	111	23	36	9	58	198	1131
Genera	39	3	0	5	2	2	12	4	1	5	2	6	3	84
Species	44	10	0	4	2	2	26	4	6	8	3	11	1	121
Unidentified	227	0	22	6	0	0	15	0	0	0	0	1	0	271
Trophic lifestyle														
Parasitic	320	20	0	5	9	12	63	111	8	36	5	58	182	829
Putative parasitic	15	0	22	2	0	2	6	0	15	0	4	0	16	82
Facultative parasitic	34	0	0	0	0	0	0	0	0	0	0	0	0	34
Putative facultative parasitic	161	0	0	25	0	0	0	0	0	0	0	0	0	186
rRNA genes														
18S rRNA	147	13	14	6	1	5	45	110	23	31	8	34	71	508
ITS	124	1	4	6	0	2	10	0	0	0	0	0	19	166
28S rRNA	189	2	4	13	4	2	14	0	0	0	1	22	12	263
18S + ITS	1	0	0	3	1	0	0	0	0	1	0	2	86	94
ITS + 28S	13	0	0	0	0	1	0	0	0	2	0	0	10	26
Complete	57	4	0	3	3	4	0	1	0	2	0	0	0	74
Hosts														
Diatoms	230	0	18	0	0	0	25	2	8	2	4	0	0	289
Dinoflagellates	36	0	0	0	0	0	0	0	0	0	0	44	196	276
Other microalgae	45	19	0	10	9	14	0	0	0	0	0	0	0	97
Macroalgae	19	0	0	1	0	0	39	10	0	21	0	0	0	90
Plants	46	0	4	8	0	0	2	99	0	13	0	0	0	172
Unknown	154	1	0	13	0	0	3	0	15	0	5	14	2	207
Habitat														
Freshwater	382	11	22	19	9	7	12	0	0	0	0	0	0	462
Marine	35	0	0	0	0	0	57	111	23	35	7	58	198	524
Industrial	5	9	0	0	0	7	0	0	0	0	0	0	0	21
Unknown	108	0	0	13	0	0	0	0	0	1	2	0	0	124

**FIGURE 4:**
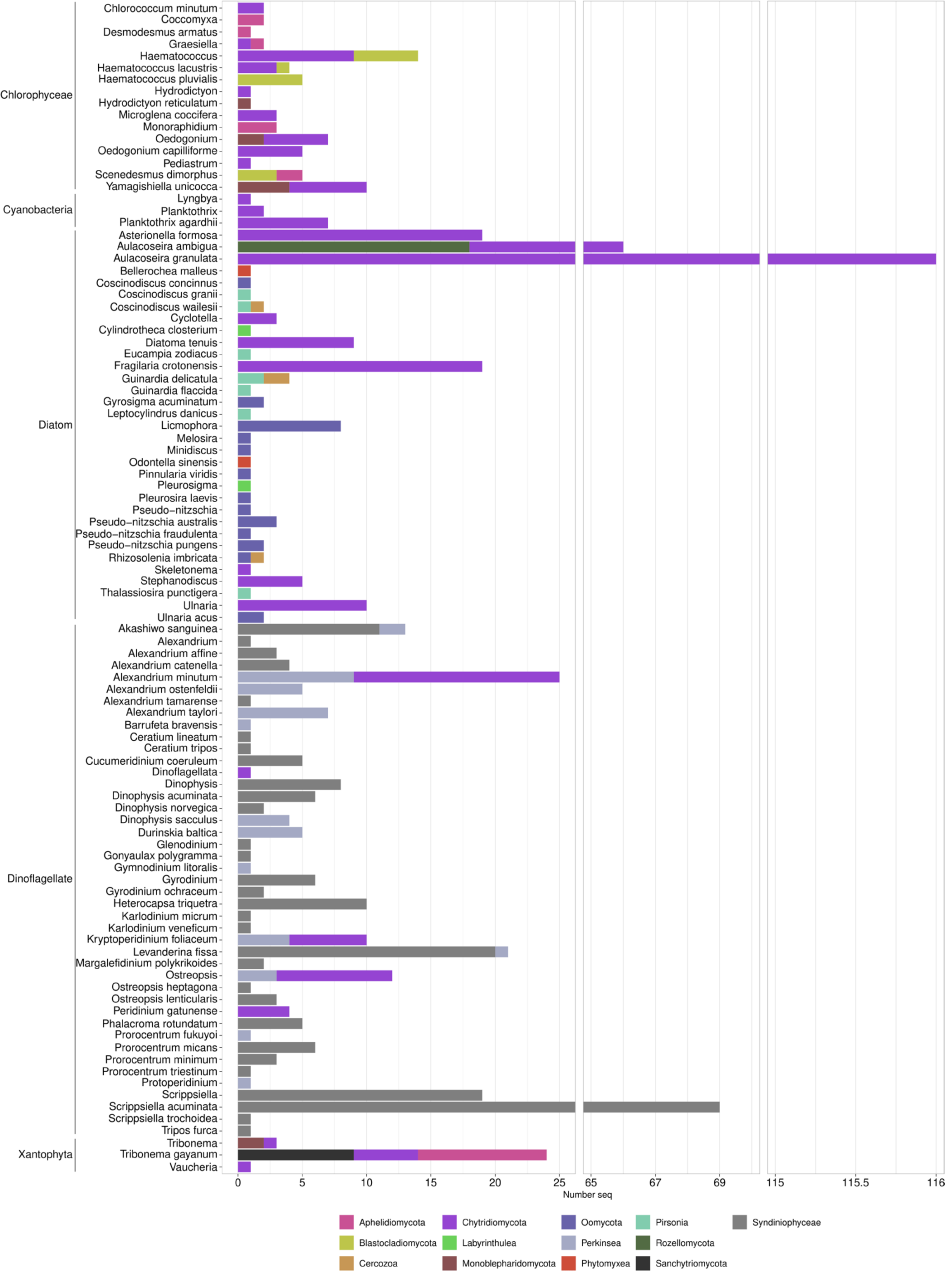
Number of sequences obtained for photosynthetic microorganism host species represented in ParAquaSeq, showing the parasitic group to which they belong The *x* axis represents the total number of sequences belonging to parasites infecting a given host species, which are represented on the *y* axis.

**FIGURE 5:**
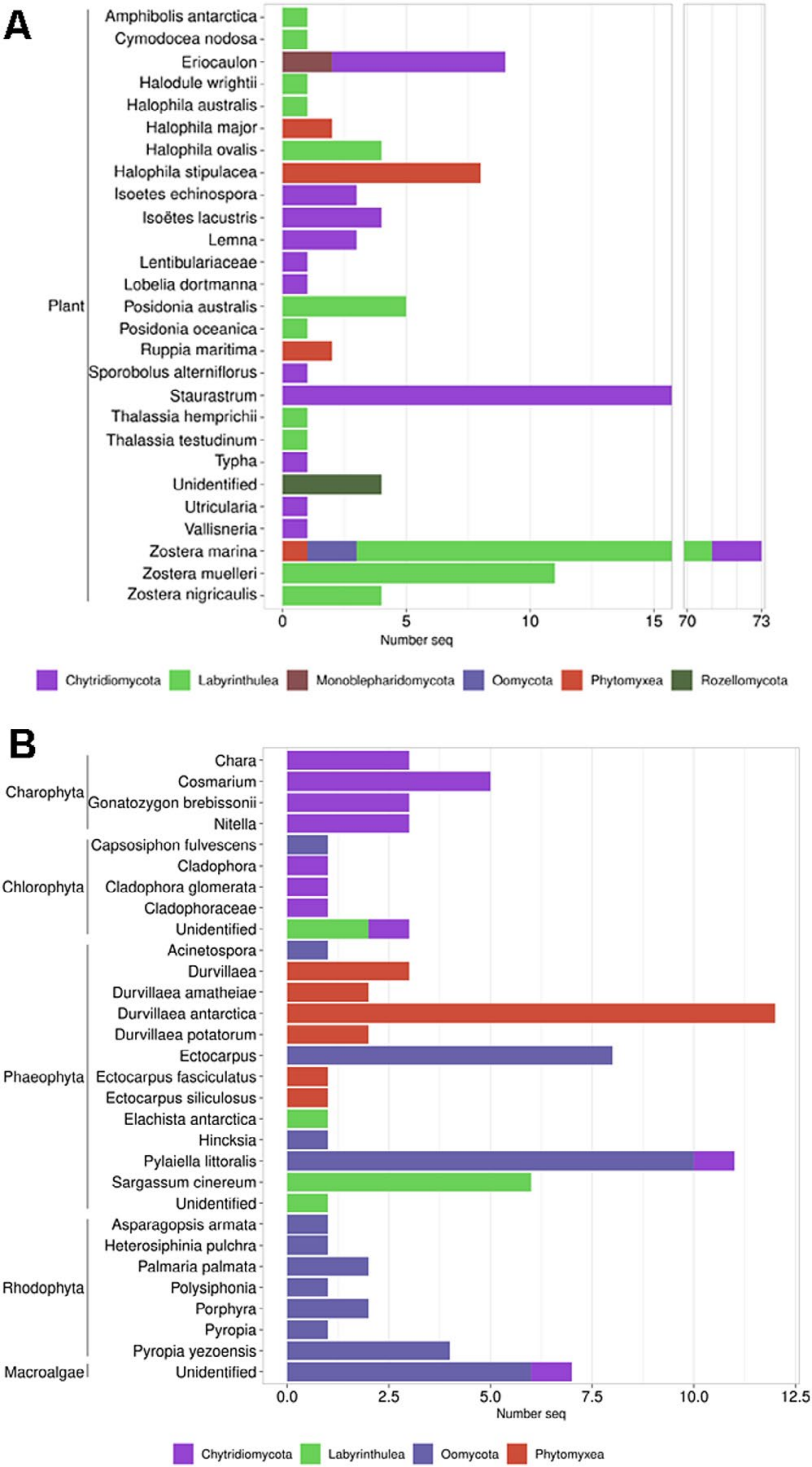
Number of sequences obtained for host species represented in ParAquaSeq, showing the parasitic group they belong to. (A) Aquatic plant hosts; (B) macroalgae hosts.

Regarding their hosts (Figure 4), most parasites detected were found to infect dinoflagellates (276 sequences) and diatoms (289 sequences), with a majority of 196 dinoflagellate parasite sequences corresponding to *Amoebophrya* spp. (Figure 3; Table 1). Other microalgae host groups like Xanthophyceae or Chlorophyceae (Figure 4) were represented by a lower number of associated parasite sequences (97). Parasites infecting macrophytes comprised 172 sequences infecting aquatic plants and 90 sequences infecting macroalgae (Figure 5A,B). Importantly, a large number of sequences lacked information about the primary host (207). Most of them corresponded to sequences classified as “putative” parasites, as they belonged to species known to be parasites of algae, but the parasitic interaction was not confirmed in the associated publication. Up to 154 of these sequences corresponded to Chytridiomycota (Figure 3).

Regarding the habitats (Figure 3; Table 1), the sequences corresponded to parasites detected in all aquatic environments, including freshwater, brackish, marine habitats, and artificial and industrial systems. Parasitic interactions were also reported from artificial and industrial systems, mainly corresponding to freshwater microalgal cultures. Altogether, 382 sequences were obtained from freshwater environments, mostly belonging to Chytridiomycota, other fungal groups, and Oomycota. Sequences from marine habitats were more diverse, being represented by all parasite groups except basal fungi, i.e., Aphelidiomycota, Monoblepharidomycota, Sanchytriomycota, Rozellomycota, and Blastocladiomycota. Parasites obtained from industrial facilities were represented by 21 sequences, only corresponding to Chytridiomycota, Aphelidiomycota, and Blastocladiomycota. Finally, the exact origin of 124 sequences was not specified in the NCBI metadata or associated bibliography; in this case, they mainly corresponded to Chytridiomycota specimens.

### 3.2 Chytridiomycota

A list of 530 Chytridiomycota ribosomal sequences from NCBI was obtained after verification and curation. The 28S rRNA was the most abundant gene region covered (present for 259 entries), followed by 18S rRNA (present for 205 entries) and ITS (present for 195 entries). There were 57 long‐read rRNA sequences available, covering the 18S, ITS, and 28S rRNA genes, mainly derived from nanopore sequencing. Out of these 530, 354 sequences belonged to confirmed parasites, both obligate (320) and facultative (34), known to have both parasitic and saprotrophic lifestyles. The remainder of the sequences (176) were classified as putative parasites (15) and putative facultative parasites (161). The majority of confirmed parasite sequences were derived from uncultured taxa obtained from environmental single‐cell isolates (185).

The database contains 71 sequences with a specimen voucher reference and 61 with a strain reference. However, a culture collection was only specified for 39 sequences out of the 530 sequences, all of which were identified at the species level, 6 corresponding to confirmed parasites, 9 to facultative parasites, and 24 to putative facultative parasites.

The majority of the confirmed Chytridiomycota parasite taxa were microalgae parasites (Table 1). They were associated with 30 different host taxa from all major microalgal groups, including diatoms, green algae, dinoflagellates, and cyanobacteria (Figure 4). Bacillariophyceae (diatoms) was the most taxon‐rich host group. Almost all diatom host taxa in the database were planktonic freshwater species, such as *Asterionella formosa* , *Fragilaria crotonensis* , *Ulnaria* sp. (formerly *Synedra* sp.), *Cyclotella* sp., *Aulacoseira ambigua* , and *A. granulata* , with the latter being the most represented host genus. Only one uncultured Rhizophydiales chytrid sequence was available from a marine diatom host species (*Skeletonema* sp.). A contrasting pattern was observed in dinoflagellates, where almost all host species were marine and brackish (e.g., *Ostreopsis* sp., *Kryptoperidinium foliaceum* , and *Alexandrium minutum* ), with only a single freshwater dinoflagellate host species ( *Peridinium gatunense* ). The database contains several sequences of formally described chytrid species infecting Chlorophyceae. The order Chlamydomonadales contained species such as *Yamagishiella unicocca* (Volvocaceae) that were hosts to several described chytrid species like *Dangeardia mamillata*, *Algomyces stechlinensis*, and *Volvorax ingoldii* (Van den Wyngaert et al. 2018), and the industrially important microalgae *Haematococcus lacustris* , host to *Quaeritorhiza haematococci*. The order Desmidiales (Desmidiaceae) included genera such as *Staurastrum* that also hosted different chytrids like *Protrudomyces lateralis* or *Staurastromyces oculus* (Van den Wyngaert et al. 2017, 2022). Furthermore, *Tribonema gayanum* was the only Xanthophyta species serving as a host for confirmed chytrid parasite species *Gromochytrium mamkaevae* and *Apiochytrium granulosporum* (Karpov et al. 2014), whereas a putative parasitic interaction was found for *Vaucheria* and the facultative parasite *Aquamyces chlorogonii*. There was only one cyanobacteria genus, *Planktothrix* spp., in the database that was host to a confirmed parasite species, presumably but not formally confirmed, *Rhizophydium megarrhizum*. Other host groups were represented, but only by a single occurrence. Finally, several sequences of confirmed parasites were associated with Charophyta, and one, *Algochytrops polysiphoniae*, former name *Chytridium polysiphoniae* , was associated with the brown macroalga *Pylaiella littoralis* . However, 31 additional chytrid sequences were recognised as putative parasitic/putative facultative parasites of aquatic macrophytes—both vascular and algae (*Chara* sp., *Eriocaulon* sp., *Isoetes echinospora* , *I. lacustris* , *Lemna* sp., *Lobelia dortmanna* , *Nitella* sp., *Typha* sp., *Utricularia* sp., *Vallisneria* sp., *Zostera marina* ).

The confirmed parasite sequences were obtained from a variety of ecosystems and habitats, including natural and aquaculture systems, freshwater and marine environments, and different types of habitats (e.g., ponds, lakes, rivers, coastal, harbour etc). Most of the parasite taxa were isolated, however, from natural freshwater lake systems. Only five sequences were obtained from freshwater industrial aquaculture, and 35 sequences were generated from coastal marine/brackish habitats.

Most of the species available in public culture collections were classified as putative parasites or confirmed facultative parasites. The largest collection is the Collection of Zoosporic Eufungi at the University of Michigan (CZEUM), which has merged the previous culture collections of the University of Maine Culture Collection (UMCC, or JEL) and the University of Alabama Chytrid Culture Collection (UACCC). Only a few confirmed obligate parasites were available in CCAP or CALU culture collections, including the species *Zygorhizidium affluens, G. mamkaeva*, and *Mesochytrium penetrans*.

### 3.3 Other Basal Fungi: Blastocladiomycota, Monoblepharidomycota, Sanchytriomycota, Aphelida, and Rozellomycota

A total of 14 sequences of Blastocladiomycota belonged to algal parasites. Despite the low number of sequences, they covered the 18S, 28S rRNA, and ITS genes, including four sequences covering the complete ribosomal region. The sequences available exclusively belonged to *Paraphysoderma sedebokerense* (11 entries) and *Catenaria* sp. (3 entries). *P. sedebokerense* was a parasite of the Chlorophyceae *Haematococcus* ( *H. pluvialis* and *H. lacustris* ) and *Scenedesmus dimorphus* . The remaining three sequences belonging to *Catenaria* sp. were also found to infect *Haematococcus* sp. All parasites were isolated in green microalgal cultures from industrial facilities like raceways or outdoor ponds. *P. sedebokerense* isolate FD61 was deposited in the Chytrid Culture Collection of the University of Alabama (UACCC), now CZEUM.

Nine sequences belonging to the recently established fungal phylum Sanchytriomycota were obtained from NCBI. They covered all ribosomal genes and represented two species, *Sanchytrium tribonematis* and *Amoeboradix gromovi*. In both cases, they were associated with the Xanthophyta *T. gayanum*, even though the latter was also found to infect *T. vulgare* and the chlorophyte *Ulothrix tenerrima* (Karpov et al. 2018). Currently, these parasites have only been detected in freshwater ditches from Russia and Finland (Karpov, Mamanazarova, et al. 2017; Karpov et al. 2018).

Monoblepharidomycota were represented by 32 sequences belonging to five different genera. Thirteen sequences corresponded to the 28S rRNA gene, but sequences including the other ribosomal genes were also present, including three complete sequences. Most sequences corresponded to putative facultative parasites, but five sequences of Polyphagales, including *Polyphagus parasiticus*, a parasite of the Xanthophyta *T. gayanum*, belonged to confirmed parasites (Karpov et al. 2016). Additionally, different Chlorophyceae genera and aquatic plants served as hosts of putative facultative parasites of the group. All sequences were obtained from freshwater environments, mostly from North American ponds and lakes.

In Aphelida, 20 sequences corresponding to algal parasites were available in NCBI. Most sequences corresponded to the 18S rRNA gene, while only one represented the ITS region and two the 28S rRNA. Finally, four sequences covered the complete ribosomal region. The sequences represented several different species belonging to three genera, *Aphelidium*, *Paraphelidium*, and *Amoeboaphelidium*. The sequences corresponded to species mostly infecting Chlorophyceae. Specifically, *Amoeboaphelidium* sp. WZ01 was associated with *Graesiella* sp., and *A. occidentale* and *A. protococcarum* with *S. dimorphus* ; *Desmodesmus armatus* hosted *Aphelidium desmodesmi, Monoraphidium* sp. hosted *A. parallelum*, and *Coccomyxa* sp. was found to be infected by *A. collabens* (Letcher et al. 2013, 2015, 2017; Ding et al. 2018; Seto et al. 2020). Sequences of parasites infecting the Xanthophyta *T. gayanum* were also present, i.e. *A*. *aff. melosirae* (Karpov et al. 2014), *A. insulamus* (López‐García et al. 2020), *A. arduennense* (Tcvetkova et al. 2019), *Paraphelidium tribonemae* (Karpov, Tcvetkova, et al. 2017; Karpov et al. 2019) and *P. letcheri* (Karpov, Torruella, et al. 2017). These organisms were mostly isolated from freshwater artificial and industrial outdoor ponds, photobioreactors, and raceways, but some of them were obtained from natural environments in Russia.

Of the almost 400 Rozellomycota sequences available in NCBI, only 22 were included in ParAquaSeq. Fourteen sequences included the 18S rRNA gene, four of them the ITS region, and four represented the 28S rRNA gene. All sequences belonged to undetermined species. Eighteen sequences corresponded to algal parasites without confirmed parasitic interactions, as they were obtained from the cell surface of diatoms isolated from a lake in Japan. Lastly, four sequences belonging to putative parasites of aquatic plants were detected in a lake from the US.

### 3.4 Oomycota

Although there were thousands of Oomycota sequences in NCBI, our search only yielded 69 entries that could be attributed to parasites of algae or aquatic plants. The sequences mostly corresponded to the 18S rRNA gene (45 entries), but also to ITS (10 entries) and 28S rRNA (14 entries).

The generic affiliation of Oomycota species has undergone many changes lately, e.g. Buaya et al. (2020), Zuccarello et al. (2024), but the sequences obtained currently belong to 12 genera, representing up to 26 species. Additionally, 15 sequences corresponded to uncultured organisms of unknown affiliation. Among them, 42 referred to oomycetes parasitising macroalgae (mostly Phaeophyceae and Rhodophyceae but also one Chlorophyta host). Eighteen sequence records belonged to *Eurychasma dicksonii* , a known parasite of brown algae, in particular of filamentous algal genera such as *Pylaiella* and *Ectocarpus*. *Pylaiella* was also the only genus identified as a host for more than one parasite species ( *E. dicksonii* , *Anisolpidium rosenvingii* and *Pontisma blauvikense*). Seaweeds belonging to red algae (representatives of genera *Porphyra*, *Polysiphonia*, *Palmaria* and *Pyropia*) were recognised as hosts of intracellular oomycetes such as *Olpidiopsis muelleri*, *Olpidiopsis palmariae* (Buaya et al. 2023; Zuccarello et al. 2024), as well as filamentous oomycetes such as *Pythium porphyrae*. A sequence derived from a parasite of the green algae *Capsosiphon fulvescens* was believed to correspond to *Sirolpidium bryopsidi*s, a known parasite of *Bryopsis plumosa*. Six additional ITS sequences belonging to *Saprolegnia* spp. and *Phytopythium litorale* were obtained in freshwater macroalgae, even though the parasitic interaction was not confirmed and the host algal species was not given. Two sequences belonging to the genera *Phytophthora* and *Halophytophthora* corresponded to parasites of the seagrass genus *Zostera*.

The remaining sequences referred to parasites of diatoms, six from freshwater environments (*Diatomophtora gillii*, *Miracula moenusica*, *M. einbuarlaekurica*, and *Aphanomycopsis bacillariacearum*) and 19 from marine ones ( *Lagenisma coscinodisci* , *Diatomophthora drebesii*, *Miracula helgolandica* and *M. islandica* and an unidentified oomycota organisms). The marine diatom hosts included the toxic, bloom‐forming diatom *Pseudo‐nitzschia pungens* (parasitised by *M. helgolandica* ) and *Rhizosolenia imbricata* (host to *D. drebesii*). The freshwater diatom hosts were represented by benthic species. In summary, 12 sequences were obtained from freshwater, while 57 entries were obtained from marine environments. The majority of them belonged to temperate regions, e.g. the North Sea, particularly the UK, and sub‐Arctic marine waters, but the exact origin of 23 entries could not be determined based on the available metadata.

### 3.5 Labyrinthulomycota

One hundred and eleven sequences available in NCBI corresponded to parasites of algae (2 diatoms and 10 macroalgae) and aquatic plants (99 entries). With only one exception that included all ribosomal genes, all sequences exclusively represented the 18S rRNA gene. The sequences mostly represented the genus *Labyrinthula*, and some representatives of the genera *Phycophthorum*, *Aplanochytrium*, and *Thraustochytrium*. The hosts were predominantly represented by *Zostera* spp., even though other aquatic plants like *Halophila ovalis* or *Posidonia* spp. were also affected by those parasites. Macroalgae parasites were mainly represented by *Thraustochytrium kinnei* sequences obtained in seaweed, brown macroalgae from the genus *Sargassum* (Damare 2015) and *Aplanochytrium* sp. infecting the brown alga *Elachista antarctica* (Mystikou et al. 2014). Sequences of unidentified *Labyrinthula* species infecting undetermined green and brown macroalgae were also reported. Finally, two sequences of *Phycophthorum isakeiti* and *Labyrinthula diatomea* were documented as parasites of the marine diatoms *Pleurosigma* sp. (Hassett 2020) and *Cylindrotheca closterium* (Popova et al. 2020), respectively. The Labyrinthulomycetes sequences were obtained from worldwide marine environments.

### 3.6 Pirsonia

Twenty‐three 18S rRNA sequences meeting the established criteria were recovered. Those sequences mostly belonged to *Pirsonia guinardiae*, but also to other species like *P. formosa* , *P. verrucosa* , *P. diadema* , *P. punctigera* and *P. chemainus*. *Pirsonia* representatives infected a large number of diatoms as hosts, e.g., *Guinardia* spp., *Coscinodiscus* spp., *Leptocylindrus danicus* or *Eucampia zodiacus* , although for 15 sequences no host was recorded. All samples came from marine environments, particularly the German North Sea.

### 3.7 Phytomyxea

The query for Phytomyxea resulted in 36 entries associated with parasites of algae and aquatic plants. Most sequences represented the 18S rRNA gene (31 entries), but one sequence included the 18S rRNA and ITS, two sequences included the ITS and 28S rRNA, and two entries comprised the three ribosomal regions. Thirteen of them corresponded to parasites of marine aquatic plants, including *Feldmanniella radicapillae* infecting *Z. marina* , *Marinomyxa* spp. infecting *Halophila* spp., and *Tetramyxa parasitica* infecting the brackish water macrophyte *Ruppia maritima* . Twenty‐one additional sequences mainly belonging to the genus *Maullinia* were associated with macroalgae, predominantly infecting the brown algae genus *Durvillaea*. *Maullinia braseltonii* was found to be associated with *Durvillaea* sp. (Murúa et al. 2017), whereas *Durvillaea antarctica* was linked to *Maullinia* sp. SN‐2012 (Goecke et al. 2012) and *M. braseltonii* (Murúa et al. 2017; Blake et al. 2017). In contrast, *M. ectocarpi* was found to infect *Durvillaea amatheiae* and *D. potatorum* (Blake et al. 2017). Only two sequences corresponded to diatom parasites, i.e. *Phagomyxa odontellae* and *P. bellerocheae*, infecting the marine diatoms *Odontella sinensis* and *Bellerochea malleus*, respectively (Bulman et al. 2001). All sequences corresponded to material obtained in marine or intertidal habitats. The exact origin of one sequence could not be determined. Preserved tissue of plants infected with *Marinomyxa* spp. was deposited in the Herbarium of the Institute of Botany, Czech Republic. Slides of *M. ectocarpii* and *M. braseltonii* were deposited at the NHM in London, UK.

### 3.8 Cercozoa: *Cryothecomonas* and *Pseudopirsonia*

Regarding *Cryothecomonas* and *Pseudopirsonia*, nine sequences were obtained from NCBI, all representing the 18S rRNA and only one the 28S rRNA gene. Seven sequences corresponded to *Cryothecomonas* species, infecting the diatoms *Guinardia delicatula* and *Thalassiosira rotula* . In the case of recovered 18S rRNA *Pseudopirsonia* sequences, the organisms were initially classified as *Pirsonia* members. However, phylogenetic analyses showed they belonged to Cercozoa, representing a new genus (Kühn et al. 2004). They were described infecting *R. imbricata* and *Coscinodiscus wailesii* . However, host information was not provided for five of the sequences. All sequences were obtained from marine habitats, the organisms being mostly present in high latitudes, i.e., North Sea and Antarctica. The origin of two sequences could not be determined.

### 3.9 Alveolata: Syndiniophyceae and Perkinsea

Among sequences obtained in our search, 58 Perkinsea and 198 Syndiniophyceae sequences corresponded to algal parasites. For Perkinsea, 34 entries corresponded to 18S rRNA, 22 to 28S rRNA, and two entries included both 18S rRNA and ITS gene sequences. For Syndiniophyceae, the variety of sequence coverage was higher, including 71 18S rRNA sequences, 19 ITS sequences, 12 28S rRNA sequences, 86 sequences including the 18S rRNA and ITS, and finally 10 sequences including the ITS and 28S rRNA genes.

Perkinsea sequences were affiliated to six genera and 11 different species, with *Parvilucifera* the most speciose, and one uncultured sequence of unknown affiliation. The final list of 198 sequences for Syndiniophyceae was mostly affiliated to the genus *Amoebophrya*. An additional 13 sequences were labelled as *Euduboscquella* sp., even though 10 of them have been recently assigned to the new genus *Hobagella* (Yoo et al. 2024). The three remaining sequences probably belong to a new, yet undescribed genus.

All represented parasites of both groups exclusively infected dinoflagellates, and information available for species represented by several sequences suggests they are generalists, being able to infect multiple dinoflagellate species but showing host preferences.

All alveolates sequences were obtained from marine samples, mostly from coastal habitats including estuaries, beaches, and harbours. The sequenced specimens were isolated from a few study areas, predominantly from South Korea and the NW Mediterranean Sea for Perkinsea and from France and China for Syndiniophyceae. Many cultures of both classes can be found in the public Roscoff Culture Collection (RCC, France), and some additional strains were available in private collections, e.g., LOHABE (South Korea), ICM (Spain).

## 4 Discussion

Our extensive sequence database, created through meticulous effort and a stringent classification method, aims to connect the molecular and ecological characteristics of parasitic species that target or are strongly associated with aquatic photosynthetic organisms. In times when metabarcoding and metataxonomics have emerged as a widespread approach to determine community composition, a database like ParAquaSeq is of high value to link traits with amplicon information not only for the parasite, but also for the host species.

The widespread use of molecular sequencing, e.g. metabarcoding, has allowed an unprecedented characterisation of environmental biodiversity, surpassing the limitations of morphological identification. It is especially relevant for microscopic organisms, whose observation and identification are not straightforward due to the frequent lack of distinctive morphological traits. However, molecular sequencing information also has important limitations, and it may not provide insights into organismal lifestyle, functional traits, and interactions of the organisms unless a well‐annotated genome is available to allow a detailed metabolic reconstruction. Nowadays, such a functional classification mostly relies on knowledge gained through literature review and available observational studies, primarily based on traditional methodologies, i.e. microscopical observations. With the aim of overcoming these limitations in broadscale assignment based on rough taxonomic classes, our database allows for a faster classification of amplicons belonging to zoosporic parasite taxa infecting aquatic primary producers.

### 4.1 Control Strategies in Commercial Production of Aquatic Primary Producers

Algal production is constantly exposed to biological constraints, challenging the production and economic viability of the activity (Carney and Lane 2014). Such contaminants include outbreaks caused by parasites, affecting seaweed cultivation (Gachon et al. 2010), green algae production (Han et al. 2013), or production of microalgae used for biofuel and farm wastewater bioremediation (Zhu et al. 2020; O'Neill and Rowan 2023). Thus, a prompt detection of parasite presence in industrial systems is required to implement rapid and efficient control strategies, thus reducing losses. ParAquaSeq allows a straightforward early detection of parasitic taxa and their close relatives in molecular datasets, providing the basis for the development of a rapid and efficient control strategy in microalgae production systems. The detection and identification of parasites allow implementing mitigation measures to minimise adverse effects of parasites in the host population. This prevents the complete crash of the entire culture and thus reduces the economic burden. As previously shown, sequences of basal fungal groups, i.e. Chytridiomycota, Blastocladiomycota and Aphelidiomycota, were directly obtained from industrial and artificial systems infecting commercially important Chlorophyceae species like *Haematococcus* spp., *Graesiella* sp. and *S. dimorphus* (Hoffman et al. 2008; Ding et al. 2018; Longcore et al. 2020). In this regard, control agents are being tested to prevent parasitic infections in these systems, e.g. the use of surfactants prevents fungal infections in *Graesiella* sp. cultures (Ding et al. 2019).

Similarly, highly economically valuable aquaculture production of red alga *Porphyra* (= *Pyropia*) is reported to be seriously damaged by infection outbreaks of oomycete pathogens *Olpidiopsis* spp. and *Pythium porphyrae* (Gleason et al. 2013; Kim et al. 2014; Badis et al. 2020). Parasites of macroalgae are being increasingly recognised as a threat to marine aquaculture, highlighting not only their potential economic impact but also the importance that a better understanding of parasite biodiversity directly translates into applied issues (Murúa et al. 2023). Consequently, it is expected that the ParAquaSeq database is adopted by industrial stakeholders as a regular tool to be used for a rapid classification and detection of parasites in metabarcoding surveys, avoiding the need to have taxonomic expertise on zoosporic parasites.

### 4.2 Biogeographical Patterns and Ecological Processes of Zoosporic Parasites

The ParAquaSeq database opens up possibilities for the meta‐analysis of zoosporic parasite occurrence and biogeography in existing and de novo generated datasets. Aquatic parasites play an important role in ecosystem functioning by increasing the bioavailability of nutrients to other organisms and in the nutrient cycles (Grami et al. 2011). They also play an important role in balancing the abundance of diverse algal host species and hence are a determinant factor of plankton community composition and aquatic food webs (Kagami et al. 2007). Furthermore, harmful algal blooms (HABs), caused by microorganisms like dinoflagellates or cyanobacteria, are subjected to parasitism, sometimes affecting their toxicity and acting as natural controlling agents (Gleason et al. 2015; Jephcott et al. 2016). Therefore, a special interest exists in determining parasitic interactions that affect aquatic communities. To detect global patterns and changes of those impacts caused by zoosporic parasites, metataxonomic approaches can only be pivotal if the sequences are lined up to knowledge on functional traits and distribution patterns. The ParAquaSeq database enables the research community to identify and fill current knowledge gaps, to better comprehend current insights into parasite and host interactions, and to advance our understanding of how changing environmental conditions may alter parasite–host interactions. Such research is of high relevance in both natural aquatic ecosystems, due to their impact on the availability of drinking water and food, as well as in artificial systems for food production.

### 4.3 Biases on Diversity Representativeness

Even though zoosporic parasites and their impacts on aquatic primary producers have been known since the early twentieth century, e.g. Canter (1950), many knowledge gaps leading to potentially biased interpretations have been evidenced when analysing the molecular sequences included in the database. A bottleneck in populating sequencing databases with new zoosporic parasite species arises from the challenge of the reliable identification of parasites, in particular specific parasite–host pairs. In the case of chytrids, most of the named species are documented in papers published before widespread culturing of taxa and molecular data existed (Canter 1950; Sparrow 1960; Voigt et al. 2021). However, there is now clear evidence that homoplasy in thallus morphology renders morphological characteristics alone inadequate indicators of phylogenetic relatedness and species identification (James et al. 2000). Therefore, connecting old names based on morphological description and their associated ecological data to newly isolated and sequenced chytrids is inherently complex and prone to identification and assignment errors. This can be partially overcome by the establishment of stable live host–parasite cultures, since cryopreservation methods are still under development. This is inherently challenging and time‐consuming, and often their maintenance relies on specialised knowledge which is linked to individual researchers, and hence scattered across different institutions and vulnerable to losses. Hence, only a few cultures are available, and the culture collections are not easily accessed. In fact, many of them are private collections, lacking a public catalogue of cultured strains, making it difficult to determine their availability and whether those strains are still alive.

The number of sequences available in NCBI for each group is highly divergent. This reflects the diversity of each taxonomic group, but also the fact that some groups have received more attention from the scientific community than others. Likewise, the available sequences for different markers are limited, since many groups are almost exclusively represented by 18S rRNA sequences. Cultivation‐independent methods, such as single‐cell sequencing, have emerged as a valuable approach for identifying and describing the diversity of algal host–chytrid associations (Ishida et al. 2015; Kagami et al. 2021; Van den Wyngaert et al. 2022; Seto et al. 2023). This advancement contributes to augmenting the number of obligate chytrid parasite sequences in molecular databases. Combining this method with long‐read sequencing can also overcome limitations of detecting parasitic species in environmental DNA (eDNA) surveys attributable to variable marker coverage (Wurzbacher et al. 2019; Seto et al. 2023).

Finally, the existence of unknown molecular diversity in several enigmatic lineages is well‐known and referred to as microbial dark matter (Rinke et al. 2013). This concept also applies to newly discovered basal fungal lineages, known as fungal dark matter, which may include zoosporic parasites (Grossart et al. 2016). For example, recent sequences of zoosporic parasites infecting the cyanobacterium *Dolichospermum* sp. resemble the morphologically described chytrid genus *Rhizosiphon*. However, their phylogenetic position in the fungal tree remains currently unresolved and cannot be assigned to any known fungal phylum (Van den Wyngaert et al. 2022; Seto et al. 2023). Consequently, these sequences are not retrieved following the taxon name searches protocol used in this study.

In fact, many entries from our initial search represented environmental sequences, with no information about the corresponding taxon or their potential parasitic nature or host. Thus, it is expected that the diversity of parasites of aquatic primary producers is much larger than currently known, and that the number of molecular sequences available for those species increases over time.

### 4.4 Knowledge Gaps on Biogeographic and Habitat Coverage

The habitat representation of sequences available differed between the parasite taxonomic groups, but the analysis of the information obtained suggests that the relatively small research community working on each parasitic group leads to the overrepresentation of a limited number of sampled systems and a low coverage of understudied regions (Frenken et al. 2017). Some groups only included algal parasites found in marine systems, e.g. Phytomyxea, Labyrinthulomycetes, Perkinsea, and Syndiniophyceae, even though none of those groups are exclusively present in marine environments, but also inhabit freshwater ecosystems. For instance, most available molecular information of Perkinsea is represented by environmental sequences obtained from freshwater environments (Bråte et al. 2010; Jobard et al. 2020; Mangot et al. 2011), but the taxa associated with those sequences and their lifestyle are unknown. The few evidences show possible infections of colonial Chlorophyceae in lakes (Mangot et al. 2013; Jobard et al. 2020). Other groups are poorly studied in regard to their functional traits, diversity, and distribution. In this case, the algal parasites present in Rozellomycota (= Cryptomycota), Blastocladiomycota, and Aphelida were exclusively detected in freshwater environments, and the sequences of the last two groups were exclusively obtained from artificial and industrial systems infecting commercial cultures. Finally, Chytridiomycota represent the most species and sequence‐rich group. Primarily driven by extensive studies on lacustrine phytoplankton‐associated chytrids (Canter 1950, 1951, 1953; Canter and Lund 1951), limnologists have predominantly explored the ecology (Van Donk and Ringelberg 1983; Ibelings et al. 2011) and taxonomy of phytoplankton parasitic chytrids (Van den Wyngaert et al. 2017, 2018; Seto and Degawa 2018; Seto et al. 2020). In contrast, only a small number of chytrid species were reported from lotic, brackish, and marine ecosystems (Sparrow 1960; Johnson and Sparrow 1961; Porter and Kirk 1987; Shearer et al. 2007). Even though sequences of Chytridiomycota parasites have a near worldwide distribution, i.e. North and South America, Europe, and Asia, a bias in sampling locations is evident since half of the parasite sequences originated from lakes in Germany and Japan. Additionally, almost all sequences from marine parasites were obtained from Mediterranean coastal sites in Spain. Therefore, this information is not a reflection of their global distribution. Nevertheless, with the growing interest in zoosporic fungi, particularly due to advancements in next‐generation sequencing, research in this area has further expanded in marine science as well (Amend et al. 2019; Grossart et al. 2019; Peng et al. 2024), resulting in an expected increase in known marine chytrid species as research continues to advance and broaden its scope (Lepelletier et al. 2014; Hassett and Gradinger 2016; Garvetto et al. 2019; Karpov et al. 2021; Fernández‐Valero et al. 2022, 2024; Reñé et al. 2022). Additionally, a few global environmental sequencing studies are available for marine environments (Grossart et al. 2019), mostly covering coastal areas from the North Atlantic Ocean and the Mediterranean Sea (Peng et al. 2024). Finally, not only biogeographic biases can be detected by the collected information, e.g. tropical and polar areas, but also many habitats remain largely unexplored, like tropical lakes, glaciers, estuaries, or benthic marine habitats. In fact, available research in polar areas has shown that sea ice is well populated with chytrids infecting diatoms (Hassett and Gradinger 2016; Kilias et al. 2020).

### 4.5 Research Needs on Host Coverage

Zoosporic parasites are known to infect multiple biotic groups, but here we focused on aquatic primary producers. Together with dinoflagellates, diatoms are the group predominantly represented as hosts in the collected sequence information, being mainly infected by chytrids. Diatoms are widespread and abundant in most aquatic systems, and parasitism on diatoms has been known since the past century (Canter and Lund 1953; Scholz et al. 2016). The lower representation of non‐chytrid diatom parasites in the database could be the result of undersampling or a general lack of research for those groups. For instance, Oomycota are well‐known parasites of diatoms too (Drebes 1966; Johnson 1966; Schnepf et al. 1978), but only a few sequences of Oomycota infecting diatoms are currently known, both from freshwater and marine environments, originating from European waters (Buaya et al. 2017, 2019, 2020, 2021; Garvetto et al. 2018). Overall, recent molecular and metabarcoding research highlights that oomycete parasites of algae are far more diverse and widespread than recognised to date (Badis et al. 2019; Hassett et al. 2019). This novel molecular knowledge is key to enable the rapid description of novel taxa, yet it remains difficult to reconcile this new knowledge with early, morphology‐based descriptions (Zuccarello et al. 2024).

Likewise, a high number of molecular sequences belonging to zoosporic parasites infecting dinoflagellates are available, mainly obtained from marine environments. Dinoflagellates are exclusively infected by Alveolata members (Syndiniophyceae and Perkinsea) and Chytridiomycota. Recently, a marine oomycete infecting dinoflagellates was described (Jeon and Park 2024). However, that sequence was not available in NCBI at the time of performing the sequence search, and it is therefore not currently included in ParAquaSeq. Moreover, freshwater dinoflagellates have been recently shown to be infected by other eukaryotic parasites (i.e., Microsporidia) (Chauvet et al. 2022).

Other microalgal groups are also infected by parasites. Sparrow (1960) described a total of 30 different cyanobacteria species being infected by different chytrids and oomycetes. However, only three chytrid sequences were found to be related to freshwater cyanobacteria in the present online database. The discrepancy between the high number of cyanobacteria species reported to be infected by chytrids and oomycetes and the low number of chytrid sequences related to freshwater cyanobacteria in current online databases may be partially explained by observed differences in infection prevalences during blooms in temperate lakes. Specifically, diatom blooms in temperate lakes are often associated with high infection prevalences (Ibelings et al. 2011; Gsell et al. 2022; Van den Wyngaert et al. 2022), whereas cyanobacteria blooms tend to show lower incidences of infections (Gsell et al. 2022; Van den Wyngaert et al. 2022) but see Rasconi et al. (2012). This can potentially lead to a higher detection and reporting of diatom‐associated chytrid sequences in current databases (however, see also our discussion above that many zoosporic parasites infecting cyanobacteria may still represent phylogenetically unresolved dark matter diversity).

Sparrow (1960) also described 16 Xanthophyta species with infections by different representatives of the Chytridiomycota. The database also includes several sequences related to confirmed parasites of xanthophytes, but infecting exclusively *T. gayanum*. Again, this reflects a lack of molecular characterisation of many parasite species, especially for those infecting microalgal groups. In contrast, unicellular Chlorophyceae are a group known to be prone to infection by fungal zoosporic parasites. In contrast to macroalgae (see below), molecular sequences collected in the database provide a good representation of current knowledge on their infective agents with no obvious biases. A reason for this might be that, basically, most Chlorophyceae hosts included are freshwater species of commercial interest, which have generally been more intensively and systematically studied.

Macroalgae and aquatic plants are foundation groups in both marine and freshwater environments and play essential roles in providing numerous ecosystem services related to habitat structuring, sediment stabilisation, primary production, oxygen production, and carbon sinks (Gleason et al. 2013; Sullivan et al. 2013). Therefore, it is vital to have access to high‐quality parasite sequences confirmed to be associated with particular hosts. A large number of sequences related to parasites of macroalgae, including Phaeophyta, Rhodophyta, Chlorophyta, or Charophyta, are represented in the database. These are mainly oomycetes and a small number of members of other parasite groups, e.g., chytrids, Phytomyxea, and Labyrinthulomycetes. Most parasite sequences associated with Rhodophyta belonged to Oomycota. Chytrid infections appeared to be more prevalent in Chlorophyta. Phytomyxea and Labyrinthulomycota predominantly infected Phaeophyta, even though they were also affected by Oomycota. Whether this is a true pattern or reflects a difference in sampling effort is difficult to judge, although the small pool of researchers contributing to data on parasitic infections indicates the latter. Although the literature recognises far more hosts and their parasites in the group of macroscopic green algae (Karling 1928 and references therein; Sparrow 1960), these reports stem from before the advent of broadscale molecular surveys. Consequently, they are not represented in ParAquaSeq. Some fungal or fungal‐like groups like Labyrinthulomycota, Phytomyxea, Chytridiomycota, or Oomycota are also well‐known pathogens of aquatic plants. Although marine macrophyte parasites are still considered understudied, *Z. marina* is the most represented species in the literature, and ParAquaSeq confirms it as a host of parasites from all above‐mentioned groups (Bockelmann et al. 2013; Govers et al. 2016; Ettinger and Eisen 2019; Kolátková et al. 2023). The other marine species are far less represented in the literature, as well as in our database. Freshwater macrophytes were also linked to the zoosporic parasites (chytrids) in ParAquaSeq, but the extent of these interactions and the possible effects of infections on natural populations are completely unknown.

## 5 Concluding Remarks

ParAquaSeq is an important resource forced by the need for fast and reliable detection of zoosporic parasites infecting aquatic primary producers in molecular datasets. At present, there is no existing tool allowing it, and the classification of the trophic lifestyle and hosts relies on the expertise of researchers. The database therefore also addresses the current diversity biases and accelerates and harmonises our knowledge of the molecular and ecological diversity of zoosporic parasites. Our work also outlines that there is a need for further development of automated high‐throughput methods such as imaging flow cytometry with automated parasite detection, coupled to sorting of parasitised algal cells and automated molecular pipelines. The scientific community can benefit from this publicly available database in multiple ways, which will help to advance aquatic research. For example, ParAquaSeq is expected to foster research and future insights into the occurrence and biogeography of these parasites and interactions with their hosts, as well as on developing detection methods for the algal biomass production industry. As such, future updates of the database, including new sequences generated or additional resources that could complement the currently produced outputs, must be considered.

## Author Contributions

Conceptioned and designed research: S.V.W., S.C., L.G., H.‐P.G., A.K., A.T., A.R. Performed research: S.V.W., S.C., L.G., A.S.G., H.‐P.G., C.L., A.K., S.N., M.S., A.T., A.R. All authors have been involved in data acquisition, drafting the manuscript, and have given final approval of the version to be published.

## Conflicts of Interest

The authors declare no conflicts of interest.

## Data Availability

All datasets generated can be accessed in GitHub, https://github.com/ParAqua‐COST/ParAquaSeq_Repository, including sequence files, metadata, explanatory documents, and scripts used to access the information. All datasets follow FAIR principles, and they are stored at Zenodo under the DOI: https://doi.org/10.5281/zenodo.14851382 (Van den Wyngaert et al. 2024).
